# Identification of Immune-Related Genes in Sepsis due to Community-Acquired Pneumonia

**DOI:** 10.1155/2021/8020067

**Published:** 2021-08-26

**Authors:** Yanyan Li, Jiqin Wang, Yuzhen Li, Chunyan Liu, Xia Gong, Yifei Zhuang, Liang Chen, Keyu Sun

**Affiliations:** ^1^Department of Emergency, Minhang Hospital, Fudan University, No. 170 Xinsong Road, Minhang District, Shanghai 201199, China; ^2^Department of Emergency Medicine, Xinhua Hospital Affiliated to Shanghai Jiaotong University School of Medicine, No. 1665 Kongjiang Road, Yangpu District, Shanghai 200092, China

## Abstract

**Background:**

Immunosuppression has a key function in sepsis pathogenesis, so it is of great significance to find immune-related markers for the treatment of sepsis.

**Methods:**

Datasets of community-acquired pneumonia (CAP) with sepsis from the ArrayExpress database were extracted. Differentially expressed genes (DEGs) between the CAP group and normal group by Limma package were performed. After calculation of immune score through the ESTIMATE algorithm, the DEGs were selected between the high immune score group and the low immune score group. Enrichment analysis of the intersected DEGs was conducted. Further, the protein-protein interaction (PPI) of the intersected DEGs was drawn by Metascape tools. Related publications of the key DEGs were searched in NCBI PubMed through Biopython models, and RT-qPCR was used to verify the expression of key genes.

**Results:**

360 intersected DEGs (157 upregulated and 203 downregulated) were obtained between the two groups. Meanwhile, the intersected DEGs were enriched in 157 immune-related terms. The PPI of the DEGs was performed, and 8 models were obtained. In sepsis-related research, eight genes were obtained with degree ≥ 10, included in the models.

**Conclusion:**

CXCR3, CCR7, HLA-DMA, and GPR18 might participate in the mechanism of CAP with sepsis.

## 1. Introduction

Recent studies have reported high mortality and morbidity in community-acquired pneumonia (CAP) [1], which is frequently complicated with sepsis. Uncontrolled excessive inflammatory response to infection and injury is a significant cause of CAP with sepsis [2]. This study shows that infection is one of the causes of sepsis [3]. CAP with sepsis is characterized by inflammatory syndrome in the early stages, which is similar to the symptoms of sepsis caused by other types of disease, such as peritonitis [4], cholangitis [5], and acute bacterial meningitis [6]. Subsequently, the body produces a series of inflammatory responses to infection, in which the immune function and the coagulation system of the body undergo complex changes [7]. It has been reported that noninvasive biomarkers for diagnosis of sepsis [8]. However, the identification of biomarkers for the sepsis diagnosis is still lacking.

The immune regulation disorder is one of the key mechanisms of sepsis. The initial immune response of sepsis is characterized by the production and release of excessive pro-inflammatory mediators, which results in clinical symptoms of fever, rapid heart rate, and shortness of breath [9]. The proinflammatory response not only is beneficial to the clearance of pathogenic agents but also increases the immune damage to the body itself [9]. An important marker of progressive sepsis is the suppression of the immune response, which is mainly manifested by the decrease in the number and function of immune cells [10], including inactivation of macrophages, low ability of antigen presentation, and decreased activity of lymphocyte proliferation. Meanwhile, the release of inhibitory cytokines is also a significant cause of induction of immunosuppression [11]. Therefore, in the pathogenesis of sepsis, immune reaction plays an important influence, which impacts the occurrence and development of sepsis. It is significant to find immune-related markers for sepsis treatment.

As a new technology, clinical bioinformatics is considered to be one of the effective methods for cancer prediction, early diagnosis, and treatment [12]. This method has been widely used in many studies, such as liver cancer and gastric cancer [13, 14]. It also plays an increasingly important role in some nontumor diseases [15]. Based on the analysis of integrating bioinformatics and meta-analysis, COL1A2 is a novel biomarker to improve clinical prediction in human gastric cancer [16]. Comprehensive bioinformatics analysis demonstrates the expression profile, clinical application significance, and prognostic value of the SLC16A gene family in pancreatic cancer [17]. In our study, we downloaded sepsis-related data from public databases to screen for differentially expressed genes. The function and pathway enrichment of different genes were delved. Based on the publication, we screened important differential genes as possible biomarkers of sepsis.

## 2. Materials and Methods

### 2.1. Data Source

From the EMBL-EBI ArrayExpress [18] database, the expression profile matrix file of preprocessed data and the standardized probe were downloaded (number: E-MTAB-5273). The chip platform was A-MEXP-2210-Illumina HumanHT-12_V4_0_R1_15002873_B. The human whole blood data of 127 community-acquired pneumonia patients with sepsis (CAP group) including 63 females and 64 males and 10 healthy subjects (normal group) including 2 females and 8 males were included in this study. Blood samples for RNA were obtained after ICU admission at the time of study enrollment (a window up to day 5). The average age of the cap group was 62.1 years, and that of the normal group was 67.9 years, and no significant difference was found (*P* = 0.25).

### 2.2. Prepreprocess of the Data

The annotation information was downloaded from the platform. Through one-to-one matching between the probe number and the gene symbol, the probes not matching the gene symbol got removed. If several probes mapped to one gene, the average value of different probes was selected as the final value of the gene, so as to get the gene expression profile.

### 2.3. Calculation of Immune Score and Screening of Differentially Expressed Genes (DEGs)

The ESTIMATE algorithm [19] was applied to estimate the immune score of 127 patients with sepsis. Based on the ESTIMATE algorithm, the immune score was calculated. Samples above the median immune score were classified as the high immune score group, while those below the median were classified as the low immune score group.

The screening of DEGs was divided into two parts. In the first part, the DEGs between the CAP group and the normal group got delved using Bayesian tests with Limma package [20] (version 3.10.3), and the *P* value was corrected by the Benjamini/Hochberg tests. The genes with ∣log fold change (FC) | >0.585 and adj. *P* value < 0.05 were identified as DEGs. Differentially expressed genes between the low immune score group and high immune score group were defined as immune-related DEGs according to adj. *P* value < 0.05 and ∣log FC | >0.585.

Furthermore, the DEGs between upregulated DEGs (CAP vs. normal group) and downregulated DEGs (high immune vs. low immune group) were selected for further analysis. Moreover, the DEGs between downregulated DEGs (CAP vs. normal group) and upregulated DEGs (high immune vs. low immune group) were also chosen for further analysis.

### 2.4. Enrichment Analysis

On the basis of the Metascape [21], intersected DEGs were performed based on Kyoto Encyclopedia of Genes and Genomes (KEGG) pathway enrichment and Gene Ontology (GO) analysis of Molecular Functions (MF), Biological Processes (BP), and Cellular Components (CC). The parameters were considered the default value. After obtaining the terms that meet the above parameters, further cluster analysis got performed based on the similarity of genes enriched in each term (similarity of >0.3). The term with the lowest *P* value in the cluster was used to represent the cluster. The top 20 clusters were retained for bar graph display. To further obtain the relationship between terms, the interactive network diagram of terms was used to show the selection criteria of terms as follows: the item with the lowest *P* value from each cluster of 20 clusters was selected, and each cluster was limited to no more than 15 items. The total number could not exceed 250 items. Terms with similarity > 0.3 were connected by edges. Finally, the interactive network of related terms was built by Cytoscape software [21] (version 3.4.0).

### 2.5. Network and Module Analysis of PPI

The Metascape was used to analyze protein-protein interaction (PPI) of intersecting DEGs based on the database of BioGrid [22], InWeb_IM [23], and OmniPath [24]. Parameters were set to default. Metascape tool based on Molecular Complex Detection (MCODE) was used for module mining in the PPI network [25]. For each module, GO BP and KEGG enrichment analysis was conducted, and the results of the top 3 of *P* value were retained for display.

### 2.6. Publication Investigation of Key Genes

The DEGs in the PPI network with a degree of connectivity ≥ 10 and the module genes mined were selected as the key genes. Based on the Biopython [26] (https://doc.yonyoucloud.com/doc/Biopython-cn/cn/chr09.html#sec-elink-citations), the sepsis-related publications were searched in the National Center for Biotechnology Information (NCBI) PubMed using the symbol of genes and sepsis as the keywords. The number of publications on sepsis of each gene was counted, and the genes corresponding to 3 ≤ number of publications ≤ 10 were taken as the focus of attention on sepsis-related genes.

To observe the gene expression in the normal group, high immune score group, and low immune score group, GraphPad Prim 5 [27] was used to draw the expression scatter diagram of each key sepsis-related gene.

### 2.7. RT-qPCR Validation

Whole human blood was added to the erythrocyte lysate, and RNA was extracted with TRIZOL (Invitrogen, USA). The total volume of the reverse transcription reaction was 20 *μ*L, including 10 *μ*L RNA, 1 *μ*L PrimeScript RT Enzyme Mix I, 1 *μ*L RT Primer Mix, 4 *μ*L 5 × PrimeScript Buffer 2, and 4 *μ*L RNase Free dH_2_O. The total volume of the quantitative real-time PCR was 20 *μ*L, including 10 *μ*L 2 × SYBR Premix Ex Taq (Takara, JPN), 0.4 *μ*L Dye II, 0.8 *μ*L PCR forward primer, 0.8 *μ*L PCR reverse primer, 2.0 *μ*L DNA, and 6 *μ*L RNase Free dH_2_O. The internal reference was GAPDH, and the primer sequences were shown in the supplementary table 1. The blood sample was selected from five sepsis patients and five normal subjects. All participants signed written consent, and this experiment got the approval of the Shanghai Minhang District Central Hospital Medical Ethics Committee (No. 2018-55).

## 3. Results

### 3.1. Differentially Expressed Analysis

**Figure 1:**
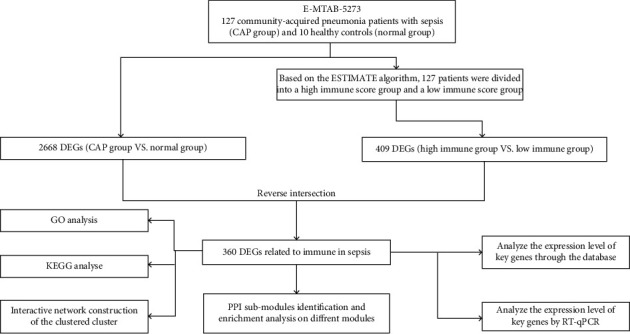
Analysis flowchart of the study.

Figure 1 shows the analysis process. According to the preprocessing analysis, there were 19,189 genes annotated. Based on the median immune score of each sample, 64 samples with high immune scores and 63 samples with low immune scores were obtained. From the three-dimensional analysis of principal component analysis (PCA) about 127 samples, the disease group and normal group could separate, which indicated that the samples could be used for further analysis (Supplementary Figure 1).

According to the screening threshold, 2668 DEGs (1052 upregulated DEGs and 1616 downregulated DEGs) were obtained between the CAP group and the normal group. Besides, 409 DEGs (238 upregulated DEGs and 171 downregulated DEGs) were obtained between the high immune score group and low immune score group.

**Figure 2:**
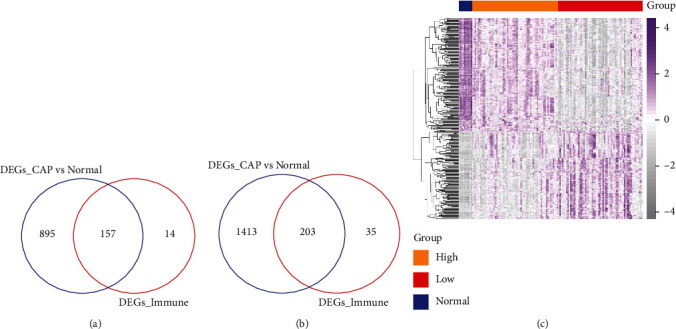
The differentially expressed genes. (a) The intersection genes of upregulated genes in community-acquired pneumonia (CAP) patients with the sepsis group and normal group with the downregulated genes in the high immune score group and low immune score group. (b) The intersection genes of downregulated genes in CAP patients with the sepsis group and normal group with the upregulated genes in the high immune score group and low immune score group. (c) Heatmap of expression of intersection genes. The expression level was indicated with the color change from gray to purple. The different color bars at the top of the heatmap represent different groups.

The intersected DEGs in two parts were obtained in the opposite direction including 360 DEGs (157 upregulated genes and 203 downregulated) as shown in Figures 2(a) and 2(b). The heatmap of the expression of intersected DEGs was shown in Figure 2(c), which indicated the significant differences among different groups of these DEGs.

### 3.2. Enrichment Analysis Result

**Figure 3:**
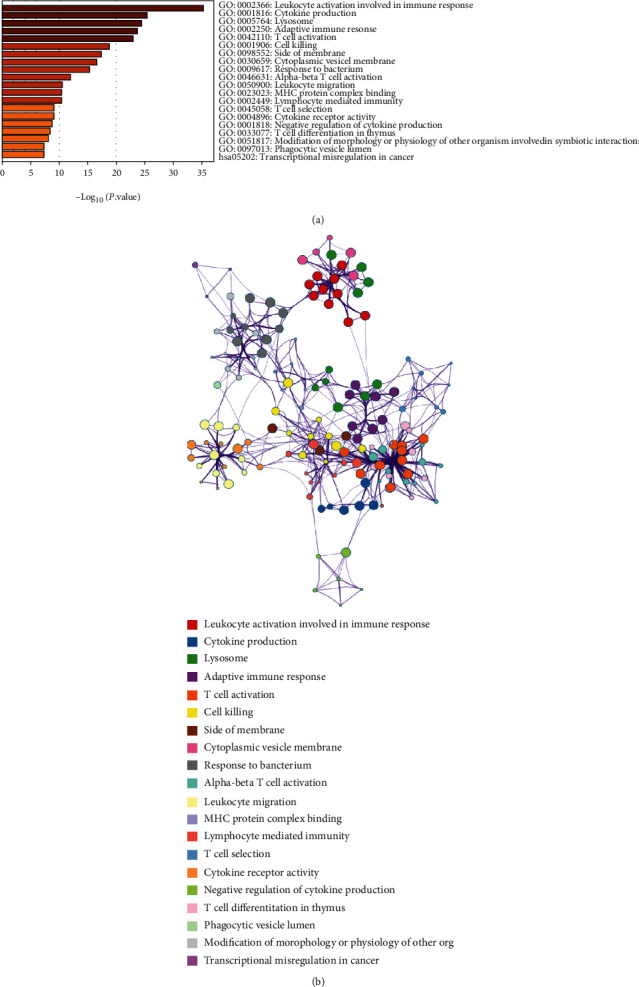
(a) The top 20 of clustering results of function and pathway enrichment analysis. The different colors represent different *P* value. (b) The interactive network diagram for enrichment analysis of functions and pathways. Different colors indicate that the enrichment results were grouped into a certain category, and the lines represented the similarity between terms.

A total of 157 enriched terms were obtained, including 122 GO BP, 15 GO CC, 9 GO MF, and 11 KEGG pathways, which were gathered in 20 clusters (Figure 3(a)). Meanwhile, the interactive network between the terms was in Figure 3(b). The data revealed the intersected DEGs were significantly enriched in the pathways which were correlated with immune, such as leukocyte activation involved in immune response, activation of the immune response, and T cell activation.

### 3.3. PPI Network and Module Analyses

**Figure 4:**
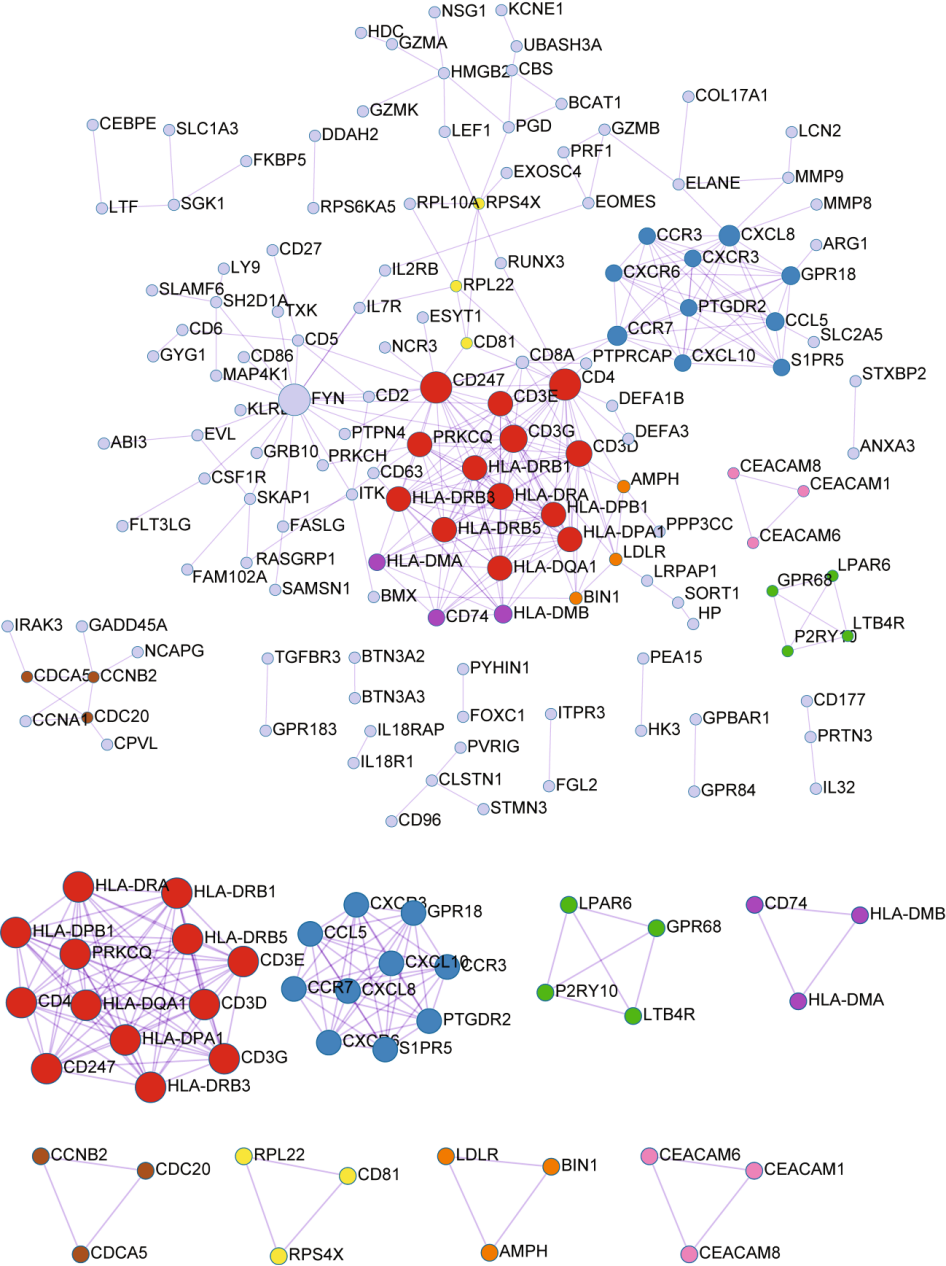
The protein-protein interaction (PPI) network and eight modules identified from the PPI network for the differentially expressed genes. Different colors indicate that the nodes belonged to different modules. The size and degree of a node were in proportion.

A total of 295 edges were obtained, including 141 protein nodes (Figure 4). The genes with node connectivity ≥ 10 were CD4 molecule (CD4), FYN proto-oncogene, src family tyrosine kinase (FYN), CD247 molecule (CD247), CD3G molecule (CD3G), major histocompatibility complex, class II, DR alpha (HLA-DRA), CD3D molecule (CD3D), major histocompatibility complex, class II, DR beta 3 (HLA-DRB3), CD3E molecule (CD3E), major histocompatibility complex, class II, DP alpha 1 (HLA-DPA1), major histocompatibility complex, class II, DP beta 1 (HLA-DPB1), major histocompatibility complex, class II, DR beta 1 (HLA-DRB1), protein kinase c theta (PRKCQ), major histocompatibility complex, class II, DQ alpha 1 (HLA-DQA1), major histocompatibility complex, class II, DR beta 5 (HLA-DRB5), C-X-C motif chemokine ligand 8 (CXCL8), C-C motif chemokine receptor 7 (CCR7), C-C motif chemokine ligand 5 (CCL5), major histocompatibility complex, class II, DM beta (HLA-DMB), and G protein-coupled receptor 18 (GPR18). Based on the MCODE algorithm, eight modules were identified (Figure 4). Enrichment analysis of GO BP and KEGG pathway was carried out for the eight modules. The top 3 results of each module are shown in Table 1.

### 3.4. Publication Search of Key Genes

**Figure 5:**
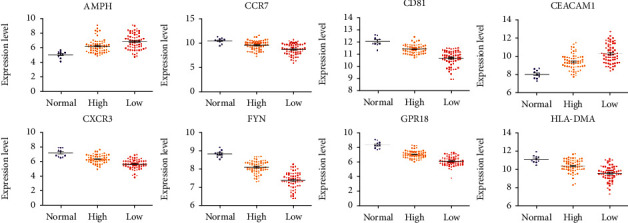
Expression of eight genes in the normal group, high immune score group, and low immune score group.

A total of 43 candidate genes with the degree ≥ 10 and included in the eight modules were taken as the key genes, which were searched for the publication on sepsis. Finally, eight genes were selected, including CCR7, C-X-C motif chemokine ligand 3 (CXCR3), FYN, CEA cell adhesion molecule 1 (CEACAM1), CD81 molecule (CD81), amphiphysin (AMPH), major histocompatibility complex, class II, DM alpha (HLA-DMA), and GPR18. The expressions of the eight genes in the normal group, high immune score group, and low immune score group are in Figure 5. AMPH and CEACAM1 had upregulated genes, which had lower immune scores corresponding to a higher expression level. The other genes were downregulated, which had the higher immune score corresponded to the higher expression level.

### 3.5. Validation of the Eight Genes

**Figure 6:**
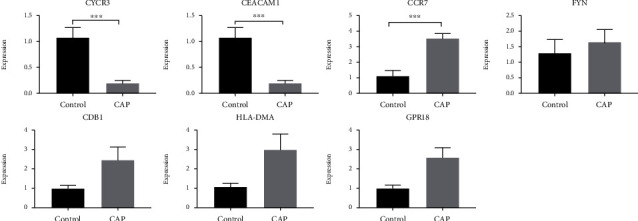
Relative expression level of CXCR3 (a) and CEACAM1 (b) in normal blood samples (control) and the blood of patients with CAP with sepsis (experiment).

We used RT-qPCR to detect the expression level of eight genes screened from 43 key genes in normal blood samples and the blood of patients in the CAP group (AMPH has not been successfully determined). As shown in Figure 6, the relative expression levels of CXCR3, HLA-DMA, CCR7, and GPR18 of the CPA group were markedly lower than those of the control group (*P* < 0.05). Compared with that in the control group, CEACAM1 in the experimental group was increased, while FYN and CD81 were decreased, with no significant difference (*P* > 0.05).

## 4. Discussion

At present, patients infected with sepsis may suffer from multiple system damage. Such as circulatory, digestive, and blood damage. Septic shock, liver abscess, heart failure, respiratory distress syndrome, etc. Immunosuppression is the main cause of death in patients with sepsis. However, the identification of biomarkers for the diagnosis of sepsis is still lacking. In this study, the enrolled samples were divided into two groups (64 samples with high immune scores, 63 samples with low immune scores). After the VENN analysis between the two groups with opposite directions, 157 upregulated genes and 203 downregulated genes were obtained. Besides, the coexpressed DEGs were enriched in 157 terms. Furthermore, 295 PPI edges were obtained which including 141 protein nodes. Through searching the sepsis-related publication of the key genes, CCR7, CXCR3, FYN, CEACAM1, CD81, AMPH, HLA-DMA, and G protein-coupled receptors (GPR18) were considered to be key genes.

Enrichment analysis reveals that genes are mainly enriched in pathways that are correlated with immune, such as leukocyte activation involved in immune response and activation of the immune response. The mechanism of sepsis and its associated systemic inflammatory response syndrome are complex. With the impaired immune function of phagocytes, immune suppression, and complement activation, a hyperinflammatory state is formed, and eventually, septic shock is triggered [28]. In conclusion, sepsis-induced organ dysfunction can be attributed to the interaction between the initial inflammatory response and the subsequent anti-inflammatory response [29].

CXCR3 belongs to the G-protein coupled seven-subunit transmembrane receptor. The expression of the gene *in vivo* is located in parenchymal cells and inflammatory cells during the lesions of multiple organs, such as blood vessels dermal cells, activated lymphocytes, macrophages, and dendritic cells [30]. CXCR3 is served as a symbol for the activity of Th1 lymphocytes [31]. Studies have shown that CXCR3 and the ligands have significant roles in the oncogene of viral infection, autoimmune disease, tumor, transplant immunity, organ/tissue fibrosis, and other diseases [32]. The related axis might be through the chemotactic activation of T cells, tumor cells, and inhibition of angiogenesis [32]. The studies also show the potential of CXCR3 blockade as a therapeutic approach to decrease the severity of sepsis during its acute phase [33].

CCR7 belongs to G protein-coupled receptor family. CCR7 and its ligands are mainly involved in the homing of T cell subsets and antigen-presenting dendritic cells to lymph nodes [34], and a previous study shows that dendritic cells and macrophages play a critical role in the inflammation of eosinophilic pneumonia [35]. CCR7 is inversely related to sequential organ failure and mortality [36].

HLA-DM, encoded by HLA-DMA and HLA-DMB, is a heterodimeric molecule that is important for normal antigen presentation. The study found that the low level of PDE4D expression was associated with HLA-DMA and HLA-DMB, while PDE4D expression continued to decline over time in sepsis patients [37].

GPR18 belongs to the orphan class A family. Studies report that modulation of GPR18 has been associated with immunomodulation, cancer, or metabolism [38, 39]. GPR18 is also involved in bacterial clearance and survival in microbial sepsis. Moreover, a previous study reveals that GPR18 plays a great role in controlling infectious inflammation and promote organ protection [40, 41]. A decrease in the positive rate of *GPR18* in neutrophils often predicts more severe sepsis and a poorer prognosis [42].

This study has some limitations. First of all, the mechanism of action of key genes in sepsis should be investigated. Secondly, we only analyzed the expression levels of seven key genes in five sepsis patients. In future studies, we will collect more clinical samples to explore the mRNA and protein levels of key genes and further study the specific role of these genes in sepsis.

In conclusion, a series of bioinformatics analyses are conducted with immune-related genes in CAP with sepsis. We obtained 360 intersected DEGs (157 upregulations and 203 downregulations). The intersected DEG was rich in 157 immune-related terms. We constructed DEG's PPI network and obtained 8 models. CXCR3, CCR7, HLA-DMA, and GPR18 might be correlated with the sepsis mechanism. These immune-related genes may play a key role in the development of sepsis and help improve the immunomodulatory treatment of patients with sepsis.

## Figures and Tables

**Table 1:** Top 3 enrichment analysis results of functions and pathways of 8 submodules.

MCODE	GO	Description	Log10 (*P*)
MCODE_1	hsa04658	Th1 and Th2 cell differentiation	-31.8
MCODE_1	hsa04659	Th17 cell differentiation	-30.9
MCODE_1	GO:0050852	T cell receptor signaling pathway	-27.2
MCODE_2	GO:0070098	Chemokine-mediated signaling pathway	-15.1
MCODE_2	GO:1990869	Cellular response to chemokine	-14.8
MCODE_2	GO:1990868	Response to chemokine	-14.8
MCODE_3	GO:0007200	Phospholipase C-activating G protein-coupled receptor signaling pathway	-6.6
MCODE_3	hsa04080	Neuroactive ligand-receptor interaction	-5.2
MCODE_4	GO:0042613	MHC class II protein complex	-9.6
MCODE_4	GO:0023026	MHC class II protein complex binding	-9.6
MCODE_4	GO:0023023	MHC protein complex binding	-9
MCODE_5	hsa04144	Endocytosis	-5.9
MCODE_6	GO:0005925	Focal adhesion	-5.3
MCODE_6	GO:0005924	Cell-substrate adherens junction	-5.3
MCODE_6	GO:0030055	Cell-substrate junction	-5.3
MCODE_7	GO:0044772	Mitotic cell cycle phase transition	-4.8
MCODE_7	GO:0051301	Cell division	-4.8
MCODE_7	GO:0044770	Cell cycle phase transition	-4.8
MCODE_8	GO:0098742	Cell-cell adhesion via plasma-membrane adhesion molecules	-5.8
MCODE_8	GO:0030667	Secretory granule membrane	-5.7
MCODE_8	GO:0043312	Neutrophil degranulation	-5.1

## Data Availability

The datasets used and/or analyzed during the current study are available from the corresponding author on reasonable request.

## Supplementary Materials

Supplementary Materials Supplementary Figure 1: the three-dimensional display diagram of principal component analysis. Supplementary Table 1: primer sequence.

## References

[1] Zheng Y., Ning P., Luo Q. (2019). Inflammatory responses relate to distinct bronchoalveolar lavage lipidome in community-acquired pneumonia patients: a pilot study. *Respiratory Research*.

[2] Neumann P. (2000). *Lung Dysfunction in the Early Phase of Sepsis*.

[3] de Simone N., Racsa L., Bevan S. (2014). Therapeutic plasma exchange in the management of sepsis and multiple organ dysfunction syndrome: a report of three cases. *Journal of Clinical Apheresis*.

[4] Culp W. T., Holt D. E. (2010). Septic peritonitis. *Compendium: Continuing Education for Veterinarians*.

[5] Beliaev A. M., Zyul'korneeva S.'., Rowbotham D., Bergin C. J. (2019). Screening acute cholangitis patients for sepsis. *ANZ Journal of Surgery*.

[6] Yang Y., Liu G., He Q. (2019). A promising candidate: heparin-binding protein steps onto the stage of sepsis prediction. *Journal of Immunology Research*.

[7] Luo H., Li X., Cao W. (2014). Advances in the research of effects of changes in immune function, coagulation function, and metabolism due to burn sepsis on wound healing. *Zhonghua shao shang za zhi*.

[8] Hu Q., Gong W., Gu J. (2019). Plasma microRNA profiles as a potential biomarker in differentiating adult-onset Still's disease from sepsis. *Frontiers in Immunology*.

[9] Novotny A. R., Reim D., Assfalg V. (2012). Mixed antagonist response and sepsis severity-dependent dysbalance of pro- and anti-inflammatory responses at the onset of postoperative sepsis. *Immunobiology*.

[10] Song G. Y., Chung C. S., Schwacha M. G., Jarrar D., Chaudry I. H., Ayala A. (1999). Splenic immune suppression in sepsis: a role for IL-10-induced changes in P38 MAPK signaling. *The Journal of Surgical Research*.

[11] Beirasfernandez A., Thein E., Hammer C. (2003). Induction of immunosuppression with polyclonal antithymocyte globulins: an overview. *Experimental and Clinical Transplantation*.

[12] Wang X., Liotta L. (2011). Clinical bioinformatics: a new emerging science. *Journal of Clinical Bioinformatics*.

[13] Tsai S., Gamblin T. C. (2019). Molecular characteristics of biliary tract and primary liver tumors. *Surgical Oncology Clinics of North America*.

[14] Yan P., He Y., Xie K., Kong S., Zhao W. (2018). In silicoanalyses for potential key genes associated with gastric cancer. *PeerJ*.

[15] Chen L., Su W., Chen H. (2018). Proteomics for biomarker identification and clinical application in kidney disease. *Advances in Clinical Chemistry*.

[16] Rong L., Huang W., Tian S., Chi X., Zhao P., Liu F. (2018). COL1A2 is a novel biomarker to improve clinical prediction in human gastric cancer: integrating bioinformatics and meta-analysis. *Pathology Oncology Research*.

[17] Yu S., Wu Y., Li C. (2020). Comprehensive analysis of the SLC16A gene family in pancreatic cancer via integrated bioinformatics. *Scientific Reports*.

[18] Kolesnikov N., Hastings E., Keays M. (2015). ArrayExpress update—simplifying data submissions. *Nucleic Acids Research*.

[19] Yoshihara K., Shahmoradgoli M., Martínez E. (2013). Inferring tumour purity and stromal and immune cell admixture from expression data. *Nature Communications*.

[20] Benjamini Y., Hochberg Y. (1995). Controlling the false discovery rate: a practical and powerful approach to multiple testing. *Journal of the Royal Statistical Society: Series B*.

[21] Zhou Y., Zhou B., Pache L. (2019). Metascape provides a biologist-oriented resource for the analysis of systems-level datasets. *Nature Communications*.

[22] Chatr-aryamontri A., Oughtred R., Boucher L. (2017). The BioGRID interaction database: 2017 update. *Nucleic Acids Research*.

[23] Li T., Wernersson R., Hansen R. B. (2017). A scored human protein-protein interaction network to catalyze genomic interpretation. *Nature Methods*.

[24] Türei D., Korcsmáros T., Saez-Rodriguez J. (2016). OmniPath: guidelines and gateway for literature-curated signaling pathway resources. *Nature Methods*.

[25] Bader G. D., Hogue C. W. (2003). An automated method for finding molecular complexes in large protein interaction networks. *BMC Bioinformatics*.

[26] Cock P. J., Antao T., Chang J. T. (2009). Biopython: freely available Python tools for computational molecular biology and bioinformatics. *Bioinformatics*.

[27] Motulsky H. (2007). *In GraphPad Prism 5: Statistics Guide*.

[28] Bosmann M., Ward P. A. (2013). The inflammatory response in sepsis. *Trends in Immunology*.

[29] Delano M. J., Ward P. A. (2016). The immune system's role in sepsis progression, resolution, and long-term outcome. *Immunological Reviews*.

[30] Priyathilaka T. T., Oh M., Bathige S. D. N. K., de Zoysa M., Lee J. (2017). Two distinct CXC chemokine receptors (CXCR3 and CXCR4) from the big-belly seahorse _Hippocampus abdominalis_ : molecular perspectives and immune defensive role upon pathogenic stress. *Fish & Shellfish Immunology*.

[31] Tsutahara K. (2012). The blocking of CXCR3 and CCR5 suppresses the infiltration of T lymphocytes in rat renal ischemia reperfusion: 812. *International Journal of Antimicrobial Agents*.

[32] Kuroki M., Kuroki M., Kinugasa T. (2008). Association between the expression of chemokine receptors CCR7 and CXCR3, and lymph node metastatic potential in lung adenocarcinoma. *Oncology Reports*.

[33] Reckamp K. L., Burdick M. D., Strieter R. M., Figlin R. A. (2005). The importance of the CXCR3/CXCR3 ligand biological axis in metastatic renal cell carcinoma. *Cancer Research*.

[34] Förster R., Davalos-Misslitz A. C., Rot A. (2008). CCR7 and its ligands: balancing immunity and tolerance. *Nature Reviews Immunology*.

[35] Nureki S., Miyazaki E., Ishi T. (2013). Elevated concentrations of CCR7 ligands in patients with eosinophilic pneumonia. *Allergy*.

[36] Almansa R., Heredia-Rodríguez M., Gomez-Sanchez E. (2015). Transcriptomic correlates of organ failure extent in sepsis. *The Journal of Infection*.

[37] Lelubre C., Medfai H., Akl I. (2017). Leukocyte phosphodiesterase expression after lipopolysaccharide and during sepsis and its relationship with HLA-DR expression. *Journal of Leukocyte Biology*.

[38] Qin Y., Verdegaal E. M. E., Siderius M. (2011). Quantitative expression profiling of G-protein-coupled receptors (GPCRs) in metastatic melanoma: the constitutively active orphan GPCR GPR18 as novel drug target. *Pigment Cell & Melanoma Research*.

[39] Morales P., Lago-Fernandez A., Hurst D. P. (2020). Therapeutic exploitation of GPR18: beyond the cannabinoids?. *Journal of Medicinal Chemistry*.

[40] Chiang N., Dalli J., Colas R. A., Serhan C. N. (2015). Identification of resolvin D2 receptor mediating resolution of infections and organ protection. *The Journal of Experimental Medicine*.

[41] Chiang N., de la Rosa X., Libreros S., Serhan C. N. (2017). Novel resolvin D2 receptor axis in infectious inflammation. *Journal of Immunology*.

[42] Zhang L., Qiu C., Yang L. (2019). GPR18 expression on PMNs as biomarker for outcome in patient with sepsis. *Life Sciences*.

