# Supplementary material for: Identification of Immune-Related Genes in Sepsis due to Community-Acquired Pneumonia
Source: Comput Math Methods Med. 2021 Aug 26;2021:8020067. doi: 10.1155/2021/8020067 (PMC8413041; doi:10.1155/2021/8020067)
Supplement: Supplementary Materials — Supplementary Figure 1: the three-dimensional display diagram of principal component analysis. Supplementary Table 1: primer sequence. [file 8020067.f1.zip › 8020067.f2.docx]

Supplementary table 1. Primer sequence.

| Primer | Forward sequence (5’-3’) | Reverse sequence (5’-3’) |
| --- | --- | --- |
| GAPDH | AATATGATTCCACCCATGGCAAAT | CCCCACTTGATTTTGGAGGGA |
| CCR7 | AACTTTGAGCGCAACAAGGC | CAGGACCACCCCATTGTAGG |
| CXCR3 | CAGCCATGGTCCTTGAGGG | GTCTTCCAGGGCCGTACTTC |
| FYN | TTGAAACACTTCAGCAGCTTGT | TAAGCCTTGGCATCCCTTTGT |
| CEACAM1 | CCACTTCACAGAGTGCGTGTA | GGCGGGTTCCAGAAGGTTAG |
| CD81 | ATCGCCAAGGATGTGAAGCA | AGTCAAGCGTCTCGTGGAAG |
| HLA-DMA | GGGAGATGCTCCTGCCATTT | GGCTTCAGCGTGAACACTTC |
| GPR18 | ACAAAATTGCAGCCCTTGTCTT | CGTGGTTCTCTTCTTGGTGGT |
