# Supplementary figures and images for: Identification of Immune-Related Genes in Sepsis due to Community-Acquired Pneumonia
Source: Comput Math Methods Med. 2021 Aug 26;2021:8020067. doi: 10.1155/2021/8020067 (PMC8413041; doi:10.1155/2021/8020067)

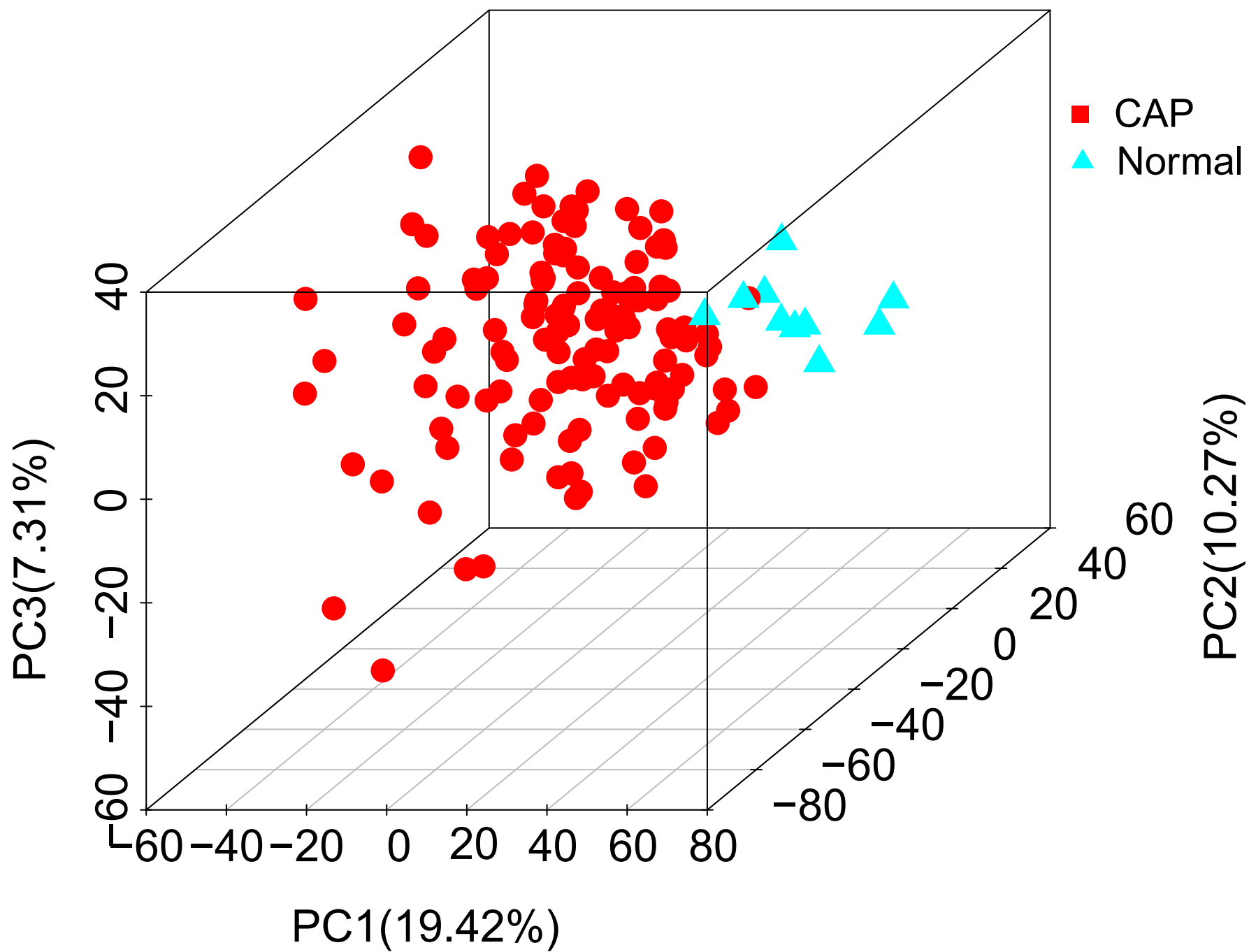

Supplement: Supplementary Materials — Supplementary Figure 1: the three-dimensional display diagram of principal component analysis. Supplementary Table 1: primer sequence. [file 8020067.f1.zip › 8020067.f1.pdf]
